# Mild to Severe Depressive Symptoms in Elderly Stroke Survivors and Its Associated Factors: Evidence From a Cross-Sectional Study in Zhejiang Province, China

**DOI:** 10.3389/fpsyt.2020.551621

**Published:** 2021-02-25

**Authors:** Xinyi Wang, Fudong Li, Tao Zhang, Fan He, Junfen Lin, Yujia Zhai, Min Yu

**Affiliations:** ^1^Department of Public Health Surveillance and Advisory, Zhejiang Provincial Center for Disease Control and Prevention, Hangzhou, China; ^2^Director Office, Zhejiang Provincial Center for Disease Control and Prevention, Hangzhou, China

**Keywords:** depressive symptom, stroke, elder, prevalence, associated factors

## Abstract

**Objective:** The objective of the study is to explore the prevalence of mild to severe depressive symptoms in elderly stroke survivors and its associated factors.

**Methods:** We did data analyses of 335 elders with stroke history. Data were collected in a survey conducted between 2014 and 2015, among permanent residents aged 60 and older in Zhejiang Province, China. Prevalence of mild to severe depressive symptoms among stroke survivors were calculated, and univariate analyses and multilevel logistic regression were used to explore its associated factors.

**Results:** Prevalence of mild to severe depressive symptoms was 22.09% (95% CI: 17.65–26.53%) in elders with stroke history, more than twice compared to their counterparts not suffering stroke (9.77%, *P* < 0.001). In multilevel logistic regression, we found that elderly stroke survivors who were illiterate (OR = 2.33, *p* = 0.008), or had limitation in activities of daily living (OR = 3.04, *p* = 0.001) were more likely to be present with mild to severe depressive symptoms, respectively, while those with more fresh vegetable consumption were at lower odds (OR = 0.82, *p* = 0.047).

**Conclusions:** Prevalence of mild to severe depressive symptoms was high in elderly stroke survivors. Targeted screening might be needed for those being illiterate, disabled in activities of daily living, and having little consumption of fresh vegetable. The association between fresh vegetable consumption and depressive symptom in stroke patients calls for further research.

## Introduction

Post-stroke depression (PSD) is the most frequent psychiatric problem in stroke survivors, with prevalence varying between populations, assessments, stages after stroke, and inclusion criteria (1). A 2014 meta-analysis stated its prevalence of 31% (2), and a 2017 one described it as 33.5%, with 17.7% in major depression, 13.1% in minor depression, and 3.1% in dysthymia (3). Meanwhile, depression was found to be an important role in many negative consequences of stroke patients. A recent review concluded that PSD was related to all-cause mortality with a hazard ratio of 1.59 (95% CI: 1.30–1.96), after analyzing data from 14 studies (4). In a large-scale, multicenter study in China, depression at 1 year after stroke was significantly associated with poor mental component summary (MCS) scores at 5 years (5). According to a research in Finland, the hazard ratio of recurrent ischemic stroke for depression group was higher than the remaining group (RR = 1.68, 95% CI: 1.07–2.63) (6). A similar association was seen between PSD and recurrent stroke at 1 year (OR = 1.49, 95% CI: 1.03–2.15) in a Chinese cohort study (7).

Stroke is estimated to increase to 23 million in 2030 (1), and PSD is expected to increase accordingly, making it an even more severe public health problem. Many factors associated with PSD were researched, such as age (8), gender, education (9), social support (10, 11), activities of daily living (ADL) (12–14), severity of stroke (15), cognitive impairment, and history of depression (16). However, majority of the previous studies were based on medical care settings (17, 18) and rarely included factors relating to post-stroke health management, such as dietary, body mass index (BMI), and blood pressure control. The current research is based on a community-dwelling elderly population, and investigated a variety of factors including sociodemographic characteristics, underlying condition, frequency of stroke, sequela of stroke, activity of daily living, cognitive function, BMI and blood pressure control, dietary, and other health behaviors. We aim to explore the prevalence of mild to severe depressive symptoms in elderly stroke survivors and its associated factors, to further provide screening indicators for depression among stroke survivors in community.

## Methods

### Data Source

Data were obtained from an investigation carried out by Zhejiang Provincial Center for Disease Control and Prevention. It was conducted between 2014 and 2015 in seven randomly selected counties/districts among a total of 90 in Zhejiang Province in eastern China. For each county/district, one or two towns/subdistricts were further selected, and all permanent residents aged 60 years old and above were invited into the survey. A written consent was obtained from each participant. For illiterate elders, informed content was read by their interviewers and signed with finger prints. The investigation was approved by the ethics committee of Zhejiang Provincial Center for Disease Control and Prevention.

Participants were interviewed face to face by trained public health practitioners and nurses using a self-designed questionnaire. The main collected information included demography, socioeconomic status, social support, underlying conditions, activity of daily living, cognitive function, reproductive history, health behaviors, and depressive symptoms. Missing data and logical errors were checked by the staff at Zhejiang Provincial Center for Disease Control and Prevention and returned to the initial interviewers who would try to reinvestigate and correct the information. In total, 10,911 elders were enrolled in the investigation, and those with a stroke history were included in the current study (*n* = 335).

### Variable of Depressive Symptom

Depressive symptom was examined by the Patient Health Questionnaire-9 (PHQ-9). Frequency of nine depressive-related symptoms in the past 2 weeks were collected for each participant, with higher frequency assigned a higher score. Total score ranges from 0 to 27, with a higher score indicating a more severe level. In this study, elders who got total scores of five and above were considered as having mild to severe depressive symptoms (19).

### Variables of Sociodemographic Information, Health Condition, and Behaviors

Gender, age, ethnicity, education, marital status, employment, perception of economic status, source of income, and having a child were included as sociodemographic factors. Perception of economic status was defined as participants' self-report of their income level in the residential areas. There were three levels to the answer, which were poor, median, and rich. Source of income was categorized into three groups, which were self-dependent only, partner-dependent only, and others. Having a child was asked by the question, “Do you have a child currently?” Never having a child and having a child pass away were recorded as no.

Frequency of stroke, sequela of stroke, underlying conditions, BMI and blood pressure control, cognitive impairment, and limitation in daily activity were included as health condition. Underlying conditions included the following diseases: hypertension, hyperlipidemia, diabetes, coronary heart disease, emphysema, pulmonary tuberculosis, asthma, chronic bronchitis, gallstone, chronic hepatitis, nephritis, tumor, Parkinson's disease, arthritis, cataract, and glaucoma. Answers to it were stratified into the following three degrees: no underlying condition, one, and two and more. Blood pressure, weight, and height were examined twice the same day as the questionnaire survey. A blood pressure gap exceeding 10 mmHg between the first and second result was considered poor quality, and a third or more test would be performed. BMI was calculated as body weight (kg)/height^2^ (m^2^). Those whose blood pressure below 140/90 and BMI between 18.5 and 24 were assessed as having their blood pressure and BMI controlled, respectively. Cognitive function was examined by the mini-mental state examination (MMSE), with a total score ranging from 0 to 30. Participants who were (1) illiterate and scored <18, (2) educated in primary school level and scored <21, or (3) educated in middle school level and above and scored <25 were defined as having cognitive impairment (20). Limitation in daily activity was investigated by the Elderly Activities of Daily Living Scale in the Chinese National Standard of Basic Public Health Service (21). Five activities were asked which were eating, bathing, dressing, toilet hygiene, and functional mobility. Participants who reported being independent to all of them were regarded as having no limitation.

Smoking, drinking, physical activity, fresh fruit and vegetable consumption, living and eating arrangement, and sedentary lifestyle were included as health behaviors. Participants were asked their behaviors in the recent year. Fresh fruit and vegetable consumption were asked by question of how frequently on average you had fresh fruit/vegetable. Categories of answers to fruit consumption were (1) barely not, (2) 1 day a week, (3) 2 days a week, (4) 3 days a week, (5) 4 days a week, and (6) 5 days and above a week. Categories to vegetable consumption's answer were (1) 2 days and fewer a week, (2) 3 days a week, (3) 4 days a week, (4) 5 days a week, (5) 6 days a week, and (6) 7 days a week. Living arrangement was asked by the question, “Usually how many persons were there living together with you (for at least half a year)?” Answers were divided into two groups: living alone and not alone. Eating arrangement was defined the same way. Sedentary lifestyle was asked by the question, “Usually how many hours do you spend each day sitting or lying except for sleep (in the past year)?”

### Data Analysis

Factors about sociodemographic information, health conditions, and behaviors were described and examined, respectively, of their univariate association with mild to severe depressive symptoms. Chi-square test, *t*-test, Chi-square test for trend, or Wilcoxon rank-sum test were used for univariate analysis, as appropriate. Exact *P*-values were calculated when necessary. In multivariate analysis, those variables showing statistical association with mild to severe depressive symptoms in univariate analyses were included. Multilevel logistic regression with random intercept was employed to explore factors associated with mild to severe depressive symptoms in elders of stroke history, taking into account county/district difference of depressive symptoms.

All analyses were performed using STATA SE 12. All statistical tests reported were two-sided, and *P* < 0.05 was considered as statistically significant.

## Results

### Prevalence of Mild to Severe Depressive Symptoms

The mean age of elders with stroke history included in the study was 72.11 ± 7.22 years old, with a gender ratio (men: women) of 1.72. Prevalence of mild to severe depressive symptoms in the participants was 22.09%, with a 95% confidence interval (95% CI) between 17.65 and 26.53%. It is significantly higher than in their counterparts without stroke history (9.77%, *P* < 0.001), according to the investigation carried out in seven randomly selected counties/districts in Zhejiang Province as mentioned in the Methods section.

### Sociodemographic Information of Study Participants

Sociodemographic information of study participants and their univariate associations with mild to severe depressive symptoms are shown in Table 1. A statistically higher percentage of illiteracy was seen in elderly stroke survivors with mild to severe depressive symptoms than those without (63.51%:43.30%, *P* = 0.002). For the rest of the variables, no statistical difference was found between the two groups (*P* > 0.05).

**Table 1:** Sociodemographic information of study participants and their univariate associations with mild to severe depressive symptoms.

**Sociodemographic** **characteristics**	**Total**		**Non-depressed** **group**		**Depressed** **group**		***X*^**2**^**	***P-*** **value**
	***N***	**%**	***N***	**%**	***N***	**%**		
**Gender**								
Men	212	63.28	172	65.90	40	54.05	3.482	0.062
Women	123	36.72	89	34.10	34	45.95		
**Age**								
60–64	54	16.12	41	15.71	13	17.57	3.614	0.461
65–69	84	25.07	70	26.82	14	18.92		
70–74	70	20.90	52	19.92	18	24.32		
75–79	65	19.40	53	20.31	12	16.22		
≥80	62	18.51	45	17.24	17	22.97		
**Ethnicity**								
Han ethnicity	321	95.82	251	96.17	70	94.59	/	0.52^#^
Minorities	14	4.18	10	3.83	4	5.41		
**Education level**								
Illiterate	160	47.76	113	43.30	47	63.51	9.446	0.002
Literate	175	52.24	148	56.70	27	36.49		
**Marital status**								
Single/divorced/widowed	72	21.49	54	20.69	18	24.32	0.451	0.502
Married	263	78.51	207	79.31	56	75.68		
**Employment**								
Working	92	27.46	76	29.12	16	21.62	1.627	0.202
Retired/never worked	243	72.54	185	70.88	58	78.38		
**Perception of economic condition**								
Rich	20	5.97	18	6.90	2	2.70	4.276	0.118
Moderate	256	76.42	202	77.39	54	72.97		
Poor	59	17.61	41	15.71	18	24.32		
Source of income^*^								
Self-dependent only	83	24.85	71	27.31	12	16.22	/	0.096^#^
Partner-dependent only	9	2.69	6	2.31	3	4.05		
Others	242	72.46	183	70.38	59	79.73		
**Currently having a child**								
Yes	328	97.91	255	97.70	73	98.65	/	1.00^#^
No	7	2.09	6	2.30	1	1.35		

* Missing data observed.

# *Fisher's exact test applied*.

### Health Conditions of Study Participants

Health conditions of study participants and their univariate associations with mild to severe depressive symptoms are shown in Table 2. There were statistically more participants having a sequela of stroke in the group with mild to severe depressive symptoms compared to their counterparts (66.67%:51.61%, *P* = 0.024). More participants were seen as having cognitive impairment in this group (40.54%:26.44%, *P* = 0.019). A much higher proportion of limitation in daily activity was observed as well, with nearly three times odds in the group with mild to severe depressive symptoms compared to those without (44.59%:14.56%, *P* < 0.001). For factors like frequency of stroke, underlying conditions, and blood pressure and BMI control, no statistical difference was found between participants with mild to severe depressive symptoms and those without (*P* > 0.05).

**Table 2:** Health conditions of study participants and their univariate associations with mild to severe depressive symptoms.

**Health condition**	**Total**		**Non-depressed** **group**		**Depressed** **group**		***X*^**2**^**	***P*-value**
	***N***	**%**	***N***	**%**	***N***	**%**		
**Frequency of stroke**								
Once	285	85.07	225	86.21	60	81.08	1.193	0.275
Twice and more	50	14.93	36	13.79	14	18.92		
Sequela of stroke^*^								
No	144	45.00	120	48.39	24	33.33	5.109	0.024
Yes	176	55.00	128	51.61	48	66.67		
**Underlying conditions**								
None	42	12.54	34	13.03	8	10.81	4.589	0.101
One	170	50.75	139	53.26	31	41.89		
Two and more	123	36.72	88	33.72	35	47.30		
**Blood pressure control**								
No	155	46.27	121	46.36	34	45.95	0.004	0.950
Yes	180	53.73	140	53.64	40	54.05		
Body mass index (BMI) control^*^								
No	299	94.32	234	93.60	65	97.01	/	0.382^#^
Yes	18	5.68	16	6.40	2	2.99		
**Cognitive impairment**								
No	236	70.45	192	73.56	44	59.46	5.509	0.019
Yes	99	29.55	69	26.44	30	40.54		
**Limitation in daily activity**								
No	264	78.81	223	85.44	41	55.41	31.140	<0.001
Yes	71	21.19	38	14.56	33	44.59		

* Missing data observed.

# *Fisher's exact test applied*.

### Health Behaviors of Study Participants

Health behaviors of study participants and their univariate associations with mild to severe depressive symptoms are shown in Table 3. A trend was seen indicating that there were more participants with higher frequency of fresh fruit consumption in the group without mild to severe depressive symptoms (*P* = 0.019). A similar pattern was observed for frequency of fresh vegetable consumption (*P* = 0.008). No statistical difference was found between the group with mild to severe depressive symptoms and those without based on factors of smoking, physical activity, drinking, living alone, eating alone, and sedentary lifestyle, respectively (*P* > 0.05).

**Table 3:** Health behaviors of study participants and their univariate associations with mild to severe depressive symptoms.

**Health behavior**	**Total**		**Non-depressed** **group**		**Depressed** **group**		***X*^**2**^/Z/T**	***P*-value**
	***N***	**%**	***N***	**%**	***N***	**%**		
**Smoking**								
Currently yes	67	20.00	56	21.46	11	14.86	3.404	0.182
Quitted	54	16.12	45	17.24	9	12.16		
Never	214	63.88	160	61.30	54	72.97		
**Physical activity**								
No	261	77.91	200	76.63	61	82.43	1.129	0.288
Yes	74	22.09	61	23.37	13	17.57		
**Drinking**								
Currently yes	57	17.01	48	18.39	9	12.16	1.766	0.414
Quitted	74	22.09	58	22.22	16	21.62		
Never	204	60.90	155	59.39	49	66.22		
**Fresh fruit consumption (days per week)**								
0	129	38.51	94	36.02	35	47.30	−2.34	0.019^$^
1	39	11.64	29	11.11	10	13.51		
2	69	20.60	56	21.46	13	17.57		
3	40	11.94	31	11.88	9	12.16		
4	11	3.28	9	3.45	2	2.70		
5 and more	47	14.03	42	16.09	5	6.76		
**Fresh vegetable consumption (days per week)**								
2 and fewer	15	4.48	10	3.83	5	6.76	−2.64	0.008^$^
3	19	5.67	13	4.98	6	8.11		
4	17	5.07	10	3.83	7	9.46		
5	31	9.25	22	8.43	9	12.16		
6	8	2.39	6	2.30	2	2.70		
7	245	73.13	200	76.63	45	60.81		
**Living alone**								
No	284	84.78	225	86.21	59	79.73	1.874	0.171
Yes	51	15.22	36	13.79	15	20.27		
**Eating alone**								
No	270	80.60	214	81.99	56	75.68	1.471	0.225
Yes	65	19.40	47	18.01	18	24.32		
**Sedentary lifestyle (hours per day)**								
Mean (standard error)	3.21 (0.13)	3.13 (0.14)	3.49 (0.30)	−1.138	0.256^&^			

$ Non-parametric test for trend applied.

& *t-test applied*.

### Multilevel Logistic Regression for Depressive Symptom

Based on the univariate analysis results, education level, sequel of stroke, cognitive impairment, limitation in daily activity, and fresh fruit and vegetable consumption were further included in multivariate analysis. Multilevel logistic regression for the association between selected factors and mild to severe depressive symptoms is shown in Table 4. In this model, association between illiteracy and mild to severe depressive symptoms still reached statistical level, with an odds ratio (OR) of 2.33 (*P* = 0.008). Limitation in daily activity remained a strong statistical association with mild to severe depressive symptoms (OR = 3.04, *P* = 0.001). Fresh vegetable consumption's statistical association with mild to severe depressive symptoms was observed as well, with higher frequency of fresh vegetable consumption relating to lower odds of mild to severe depressive symptoms (OR = 0.82, *P* = 0.047). Sequela of stroke and cognitive impairment were seen positively associated with mild to severe depressive symptoms, respectively, while higher frequency of fresh fruit consumption was seen negatively associated; however, they all failed to reach the statistical level (*P* > 0.05).

**Table 4:** Multilevel logistic regression for association between selected factors and mild to severe depressive symptoms.

**Factors**	**Odds ratio**	**95% Confidence interval**	***P*-value**
Education level(illiteracy)	2.33	(1.24, 4.35)	0.008
Sequala of stroke	1.47	(0.77, 2.80)	0.245
Cognitive impairment	1.06	(0.55, 2.04)	0.855
Limitation in daily activity	3.04	(1.54, 6.00)	0.001
Fresh fruit consumption	0.90	(0.75, 1.09)	0.278
Fresh vegetable consumption	0.82	(0.68, 1.00)	0.047

## Discussion

### Prevalence of Mild to Severe Depressive Symptoms

About one third of the patients develop depression after stroke, ranging from 23% at 5 years to 36% at 2–5 months after onset, according to a scientific statement from the American Heart Association issued in 2017 (22). It was concluded consistently that depression is one of the most common complications and the most frequent psychiatric problem among stroke survivors, though the prevalence varies greatly according to different population, assessments, stages after stroke, and inclusion criteria (1), What is more, it has not been significantly reduced in decades based on a comparison of two meta-analysis in 2005 (pooled frequency estimate: 33%, 95% CI: 29 to 36%) and 2014 (pooled frequency estimate: 31%, 95% CI: 28 to 35%), conducted by the same group of scientists (2).

In the current study, prevalence of mild to severe depressive symptoms was 22% among elderly stroke survivors in an economically developed province in China. Among previous evidence in the Chinese population, the prevalence was observed mainly between 10 and 30% (5, 7, 11, 23–26), some even high up to 40% (27–29). Many of them assessed depression at an early stage after stroke and ranged between 2 weeks and 3 months following a stroke. Meanwhile, for those studies with much higher prevalence than ours, self-designed assessments were used. One study saw a prevalence of 41% even at 3 months after a stroke, using a Chinese version of the Self-rating Depression Scale (SDS) (28). Another one diagnosed 48% of stroke patients in acute stage with PSD symptoms, using a new assessment called ZhongDa diagnostic criteria—first edition (ZD-1) suggested by 65 Chinese chief doctors (29). The gap in the percentage of depressive symptoms might mainly be attributed to different assessments and stages after stroke.

Meanwhile, we found that the presence of mild to severe depressive symptoms in elders with stroke history was more than twice compared to those without. In two middle-aged and elderly Chinese cohorts, men with a stroke history had odds of 2.2 of depressive symptoms compared to those without, and women had odds of 1.8 (30). A large-scale Danish cohort has seen four times higher depression incidence in stroke survivors than in their reference population (31). A postal mail survey in Sweden and Finland concluded that depression was 1.8 times higher in elders with a previous stroke history, and the positive association remained consistent in age, gender, and country subgroups except for the 80-year olds (32). The gap revealed a greater psychiatric disease burden of stroke survivors compared to their counterparts.

### Associated Factors of Mild to Severe Depressive Symptoms

In the current study, various factors were included for analysis, such as demography, socioeconomic status, social relation, health conditions, and behaviors. Finally, three factors remained statistically associated with mild to severe depressive symptoms, which were illiteracy, limitation in activities of daily living, and fresh vegetable consumption. Education's association with PSD was studied by some researchers, but evidence was not consistent. A 2017 review concluded that education (≤ 8 years) was positively correlated to depression within 3 months after stroke (15). A later one found that education length fewer than 8 years was related to mild depression or above but not to severe depressive symptoms or major depression. When further adjusted for sex and age, the association failed to reach the statistical level. However, education was seen again associated with PSD when measured in mean years of length (9). Physical disability was stated to be consistently associated with depression after stroke according to a 2014 review (18). Later studies in Korea, Iran, and Italia showed similar results in the association between ADL in admission and PSD, based on patients in medical care settings (12–14). This association between depression and functional deficiency are likely to be bidirectional since evidence indicates that depression was also related to worse ADL recovery (14, 33).

One associated factor rarely seen in previous evidence is fresh vegetable consumption, mainly because a few studies looked at the association between fresh vegetable and depression in stroke survivors. In terms of general population, the association gained much more attention. A 2016 review calculated a combined relative risk of 0.89 in depression comparing the highest level of vegetable intake to the lowest, and saw a similar result in a subgroup analysis of the cohort studies (34). A 2018 review performed a non-linear dose–response meta-analysis and found a statistical association between vegetable consumption and depression in cross-sectional studies but not in cohort studies. When in linear meta-regression, increased vegetable consumption was associated with decreased odds of depression in both cross-sectional and cohort studies (35). A recent large-scale study, however, concluded that association between vegetable consumption and depressive symptoms was significant in women only, after combining data from adolescents in 25 low- and middle-income countries (36). More efforts are needed to explore this association among stroke survivors since current evidence is sparse. Meanwhile, further research needs to include appropriate adjustment in the analysis (37).

### Strengths and Limitations

Our results were based on a community-dwelling population and were expected to have better generalization. Meanwhile, abundant health-related factors were included in the analysis, offering a more comprehensive understanding of factors associated with mild to severe depressive symptoms for stroke patients. Nevertheless, our evidence was constrained by the following limitations. First, the direction of the association between stroke and depressive symptom was unknown. There was a possibility that depressive symptoms might occur before a stroke, and associated factors observed in our results might not be perfectly treated as risk factors. However, they could serve well as screening indicators for depression among stroke survivors and play important roles in targeting stroke patients with mild to severe depressive symptoms in the community. Second, history of depression and stroke severity, two factors previously found to be consistently associated with depression after stroke (16, 31), was missing in the current dataset. Their contribution to the association and influence on other factors could not be analyzed. Third, time duration after stroke was not collected in the survey, and associated factors might be different between stages after stroke. However, we believe that our stroke survivors are more likely to be at a chronic stage after stroke rather than acute stage. According to previous evidence, the length of stay for stroke hospitalization in Chinese population was mainly reported as more than 10 days (38–41). In a study in Wenzhou City, which was one of our investigation cities, it was observed to be 23 days (38). What is more, for some stroke survivors, there was also the length of stay for rehabilitation and readmission. Therefore, stroke survivors in our study, who participated in the investigation in community, could probably be excluded from being at an acute stage.

In conclusion, prevalence of mild to severe depressive symptoms was high in elderly stroke survivors, nearly twice compared to those without stroke history. Screening of depressive symptoms and support might be needed for those being illiterate, disabled in activities of daily living, and having little consumption of fresh vegetable. The association between fresh vegetable consumption and depression in stroke patients calls for further epidemiology study and mechanism research to draw a clearer picture.

## Data Availability Statement

The datasets presented in this article are not readily available because data are not publicly available due to local ethical restrictions. Requests to access the datasets should be directed to Min Yu, myu@cdc.zj.cn.

## Ethics Statement

The studies involving human participants were reviewed and approved by The Ethics Committee of Zhejiang Provincial Center for Disease Control and Prevention. The patients/participants provided their written informed consent to participate in this study.

## Author Contributions

XW analyzed the data and drafted the manuscript. FL and TZ cleaned and managed the data, and critically revised the manuscript. FH and JL substantially contributed to the design of the work. YZ substantially contributed to data acquisition and quality control. MY substantially contributed to the design and supervision of the work. All authors approved the final version of the manuscript.

## Conflict of Interest

The authors declare that the research was conducted in the absence of any commercial or financial relationships that could be construed as a potential conflict of interest.

## References

[1] Villa RF Ferrari F Moretti A. Post-stroke depression: mechanisms and pharmacological treatment. Pharmacol Therapeutics. (2018) 184:131–44. 10.1016/j.pharmthera.2017.11.005 29128343

[2] Hackett ML Pickles K. Part I: frequency of depression after stroke: an updated systematic review and meta-analysis of observational studies. Int J Stroke. (2014) 9:1017–25. 10.1111/ijs.12357 25117911

[3] Mitchell AJ Sheth B Gill J Yadegarfar M Stubbs B Yadegarfar M. Prevalence and predictors of post-stroke mood disorders: a meta-analysis and meta-regression of depression, anxiety and adjustment disorder. General Hospital Psychiatry. (2017) 2017:48–60. 10.1016/j.genhosppsych.2017.04.001 28807138

[4] Cai W Mueller C Li YJ Shen WD Stewart R. Post stroke depression and risk of stroke recurrence and mortality: a systematic review and meta-analysis. Ageing Res Rev. (2019) 50:102–9. 10.1016/j.arr.2019.01.013 30711712

[5] Li LJ Yao XM Guan BY Chen Q Zhang N Wang CX. Persistent depression is a predictor of quality of life in stroke survivors: results from a 5-year follow-up study of a Chinese cohort. Chin Med J. (2019) 132:2206–12. 10.1097/CM9.0000000000000400 31436596 PMC6797138

[6] Sibolt G Curtze S Melkas S Pohjasvaara T Kaste M Karhunen PJ. Post-stroke depression and depression-executive dysfunction syndrome are associated with recurrence of ischaemic stroke. Cerebrovasc Dis. (2013) 2013:336–43. 10.1159/000355145 24193249

[7] Yuan HW Wang CX Zhang N Bai Y Shi YZ Zhou Y. Poststroke depression and risk of recurrent stroke at 1 year in a Chinese cohort study. PLoS ONE. (2012) 7:e46906. 10.1371/journal.pone.0046906 23082134 PMC3474769

[8] McCarthy MJ Sucharew HJ Alwell K Moomaw CJ Woo D Flaherty ML. Age, subjective stress, and depression after ischemic stroke. J Behav Med. (2016) 39:55–64. 10.1007/s10865-015-9663-0 26245159 PMC4724284

[9] Backhouse EV McHutchison CA Cvoro V Shenkin SD Wardlaw JM. Cognitive ability, education and socioeconomic status in childhood and risk of post-stroke depression in later life: a systematic review and meta-analysis. PLoS ONE. (2018) 13:e0200525. 10.1371/journal.pone.0200525 30011299 PMC6047794

[10] Park EY Kim JH. An analysis of depressive symptoms in stroke survivors: verification of a moderating effect of demographic characteristics. BMC Psychiatry. (2017) 17:132. 10.1186/s12888-017-1292-4 28390402 PMC5385085

[11] Wang Z Zhu M Su Z Guan B Wang A Wang Y. Post-stroke depression: different characteristics based on follow-up stage and gender-a cohort perspective study from Mainland China. Neurol Res. (2017) 39:996–1005. 10.1080/01616412.2017.1364514 28828931

[12] Park GY Im S Lee SJ Pae CU. The association between post-stroke depression and the activities of daily living/gait balance in patients with first-onset stroke patients. Psychiatry Investig. (2016) 13:659–64. 10.4306/pi.2016.13.6.659 27909458 PMC5128355

[13] Haghgoo HA Pazuki ES Hosseini AS Rassafiani M. Depression, activities of daily living and quality of life in patients with stroke. J Neurol Sci. (2013) 328:87–91. 10.1016/j.jns.2013.02.027 23522526

[14] Paolucci S Iosa M Coiro P Venturiero V Savo A De Angelis D. Post-stroke depression increases disability more than 15% in ischemic stroke survivors: a case-control study. Front Neurol. (2019) 10:926. 10.3389/fneur.2019.00926 31507525 PMC6718567

[15] Shi Y Yang D Zeng Y Wu W. Risk factors for post-stroke depression: a meta-analysis. Fronti Aging Neurosci. (2017) 9:218. 10.3389/fnagi.2017.00218 28744213 PMC5504146

[16] Ayerbe L Ayis S Wolfe CD Rudd AG. Natural history, predictors and outcomes of depression after stroke: systematic review and meta-analysis. British J Psychiatry. (2013) 202:14–21. 10.1192/bjp.bp.111.107664 23284148

[17] Hackett ML Anderson CS. Predictors of depression after stroke: a systematic review of observational studies. Stroke. (2005) 2005:2296–301. 10.1161/01.STR.0000183622.75135.a4 16179565

[18] Kutlubaev MA Hackett ML. Part II: predictors of depression after stroke and impact of depression on stroke outcome: an updated systematic review of observational studies. Int J Stroke. (2014) 9:1026–36. 10.1111/ijs.12356 25156411

[19] Kroenke K Spitzer RL Williams JB. The PHQ-9: validity of a brief depression severity measure. J General Internal Med. (2001) 16:606–13. 10.1046/j.1525-1497.2001.016009606.x 11556941 PMC1495268

[20] Zeng G. Aging of Brain and Alzheimer Disease (in Chinese). Shanghai: Shanghai Scientific and Technological Literature Press. (1995). p. 228–9.

[21] National Health and Family Planning Commission of the People's Republic of China. The National Stardard of Basic Public Health Service (2011) (in Chinese). 2011:54.

[22] Towfighi A Ovbiagele B El Husseini N Hackett ML Jorge RE Kissela BM. Poststroke depression: a scientific statement for healthcare professionals from the American Heart Association/American Stroke Association. Stroke. (2017) 48:e30–43. 10.1161/STR.0000000000000113 27932603

[23] Zhang WN Pan YH Wang XY Zhao Y. A prospective study of the incidence and correlated factors of post-stroke depression in China. PLoS ONE. (2013) 8:e78981. 10.1371/journal.pone.0078981 24260141 PMC3832506

[24] Zhang T Wang C Liu L Zhao X Xue J Zhou Y. A prospective cohort study of the incidence and determinants of post-stroke depression among the mainland Chinese patients. Neurol Res. (2010) 32:347–52. 10.1179/016164110X12656393665125 20482999

[25] Liang X Liu Y Jia S Xu X Dong M Wei Y. SIRT1: The value of functional outcome, stroke-related dementia, anxiety, and depression in patients with acute ischemic stroke. J Stroke Cerebrovasc Dis. (2019) 28:205–12. 10.1016/j.jstrokecerebrovasdis.2018.09.037 30361109

[26] Zhang N Wang CX Wang AX Bai Y Zhou Y Wang YL. Time course of depression and one-year prognosis of patients with stroke in mainland China. CNS Neurosci Therapeutics. (2012) 18:475–81. 10.1111/j.1755-5949.2012.00312.x 22672300 PMC6493639

[27] Liu R Yue Y Jiang H Lu J Wu A Geng D. A risk prediction model for post-stroke depression in Chinese stroke survivors based on clinical and socio-psychological features. Oncotarget. (2017) 8:62891–9. 10.18632/oncotarget.16907 28968957 PMC5609889

[28] Wang L Tao Y Chen Y Wang H Zhou H Fu X. Association of post stroke depression with social factors, insomnia, and neurological status in Chinese elderly population. Neurol Sci. (2016) 37:1305–10. 10.1007/s10072-016-2590-1 27120072

[29] Yue Y Liu R Cao Y Wu Y Zhang S Li H. New opinion on the subtypes of poststroke depression in Chinese stroke survivors. Neuropsychiatric Dis Treatment. (2017) 13:707–13. 10.2147/NDT.S128429 28293112 PMC5345901

[30] Liu Q Cai H Yang LH Xiang YB Yang G Li H. Depressive symptoms and their association with social determinants and chronic diseases in middle-aged and elderly Chinese people. Sci Rep. (2018) 8:3841. 10.1038/s41598-018-22175-2 29497126 PMC5832867

[31] Jorgensen TS Wium-Andersen IK Wium-Andersen MK Jorgensen MB Prescott E Maartensson S. Incidence of depression after stroke, and associated risk factors and mortality outcomes, in a large cohort of danish patients. JAMA Psychiatry. (2016) 73:1032–40. 10.1001/jamapsychiatry.2016.1932 27603000

[32] Hörnsten C Lövheim H Nordström P Gustafson Y. The prevalence of stroke and depression and factors associated with depression in elderly people with and without stroke. BMC Geriatrics. (2016) 16:174. 10.1186/s12877-016-0347-6 27717324 PMC5055663

[33] Tsuchiya K Fujita T Sato D Midorikawa M Makiyama Y Shimoda K. Post-stroke depression inhibits improvement in activities of daily living in patients in a convalescent rehabilitation ward. J Phys Therapy Sci. (2016) 28:2253–9. 10.1589/jpts.28.2253 27630408 PMC5011572

[34] Liu X Yan Y Li F Zhang D. Fruit and vegetable consumption and the risk of depression: a meta-analysis. Nutrition. (2016) 32:296–302. 10.1016/j.nut.2015.09.009 26691768

[35] Saghafian F Malmir H Saneei P Milajerdi A Larijani B Esmaillzadeh A. Fruit and vegetable consumption and risk of depression: accumulative evidence from an updated systematic review and meta-analysis of epidemiological studies. British J Nutr. (2018) 119:1087–101. 10.1017/S0007114518000697 29759102

[36] Liu MW Chen QT Towne SD Jr Zhang J Yu HJ. Fruit and vegetable intake in relation to depressive and anxiety symptoms among adolescents in 25 low- and middle-income countries. J Affect Disord. (2019) 261:172–80. 10.1016/j.jad.2019.10.007 31634676

[37] Kawada T Re. Fruit and vegetable consumption and the risk of depression: a meta-analysis. Nutrition. (2018) 45:147. 10.1016/j.nut.2017.05.013 28697855

[38] Shi LY Jin R Zheng RY Wang XT Han Z. Hospital-based study of the length of stay of stroke and its influencing factors. J Wenzhou Med College. (2008) 38:459–61. 10.3969/j.issn.1000-2138.2008.05.019

[39] Yu N Gao YR Gao YJ. The relationship between the length of stay and adverse clinical outcome among patients with acute ischemic stroke. J Brain Nervous Dis. (2015) 23:197–201. 10.3969/j.issn.1006-351X.2015.03.01110700492

[40] Li Y Liu H Wang J Li Y Yu GP Ma XM. Variable lengths of stay among ischemic stroke subtypes in Chinese general teaching hospitals. PLoS ONE. (2012) 7:e45101. 10.1371/journal.pone.0045101 23028783 PMC3461000

[41] Kang JH Xirasagar S Lin HC. Risk of adverse outcomes in patients with rheumatoid arthritis hospitalized for stroke-a cross-sectional study. Clin Rheumatol. (2018) 37:2917–26. 10.1007/s10067-018-4287-8 30209695

